# Effect of photobiomodulation therapy on wound healing and post-extraction pain management of primary molars: a randomized controlled clinical trial

**DOI:** 10.1186/s12903-026-09172-y

**Published:** 2026-07-15

**Authors:** Rowan E. Salem, Niveen S. Bakry, Reham S. Soliman

**Affiliations:** Pediatric Dentistry and Dental Public Health Department, Faculty of Dentistry, Alexandria University, 32 Champollion St. Azarita, Alexandria, 21525 Egypt

**Keywords:** Photobiomodulation therapy (PBMT), Pain assessment, Tooth extraction, Wound healing

## Abstract

**Background:**

In pediatric patients, tooth extraction frequently results in unpleasant postoperative complications, including pain, inflammation, and bleeding. These painful experiences contribute to a child’s anxiety, which may reduce his compliance with subsequent dental treatments. This study aimed to evaluate the efficacy of photobiomodulation therapy (PBMT) as an adjunctive treatment to enhance wound healing and manage post-extraction pain in children.

**Materials and methods:**

This was a double-blind, split-mouth, randomized controlled clinical trial. A total of 18 children with a mean age of 6.5 years were included; each child had one primary molar bilaterally indicated for extraction. Eligible sides were randomly assigned to either the test group (*n* = 18) receiving PBMT using a diode laser of wavelength 660 nm, an output power of 100 mW, and an application duration of 60 s (total energy of 6 J), or to the control group (*n* = 18) (placebo treatment). A one-week interval was implemented between the extraction of the first and contralateral side as a washout period to optimize post-operative comfort and function. Both groups received post-extraction instructions. Primary outcomes included wound healing, assessed by Landry, Turnbull, and Howley wound healing index, and post-extraction pain, monitored over one week using a self-reported questionnaire assessing pain, discomfort, analgesic consumption, daily activities, and jaw-function impairment. Data were analyzed using the Wilcoxon signed-ranks test, and McNemar test, with significance defined as p < 0.05.

**Results:**

At day 7, the test group demonstrated significantly superior wound healing scores (Median: 5.00) compared to the placebo group (Median: 4.00) (*P* = 0.002*). Furthermore, PBMT significantly reduced the subjective feeling of discomfort on the first evening (*P* = 0.02*). Over the one-week follow-up, the test group showed no analgesic consumption compared to 33.3% in the placebo group (*P* = 0.03*). It also reported significantly less difficulty with chewing on the extraction site, chewing soft food, and difficulty with taking a big bite compared to the placebo group; with percentages (38.9% vs.88.9%, *P* = 0.004*), (0% vs.33.3%, *P* = 0.03*), and (33.3% vs.72.2%, *P* = 0.04*), respectively.

**Conclusion:**

Photobiomodulation therapy is an effective adjunctive treatment following primary mandibular molar extraction. It significantly accelerates wound healing, reduces discomfort, and improves functional recovery by significantly reducing the need for post-operative analgesics.

**Trial registration:**

This study was prospectively registered on March 6, 2025 (Trial registration number: NCT07033403).

**Supplementary Information:**

The online version contains supplementary material available at 10.1186/s12903-026-09172-y.

## Background

In pediatric patients, tooth extraction frequently results in unpleasant postoperative complications, including pain, inflammation, and bleeding. These painful experiences contribute to a child’s anxiety and may reduce their compliance with subsequent dental treatments. Therefore, efficient prevention and management of post-extraction pain (PEP) represent a critical component of high-quality pediatric dental care [1, 2]. Wound healing and pain perception following tooth extraction are tied to several patient-related factors such as immune status, local infection, and the use of analgesics and antibiotics. Other crucial factors are how atraumatic the procedure was and how effectively post-extraction instructions were followed [3].

Assessing adjunctive therapies that can minimize child discomfort and enhance the healing process without side effects would greatly benefit pediatric dental practice. Notable innovative treatments like photobiomodulation therapy (PBMT) demonstrate considerable potential for managing inflammation, accelerating both soft tissue healing and bone regeneration, while effectively alleviating post-operative discomfort [4].

Photobiomodulation therapy, known previously as Low Level Laser Therapy (LLLT), is a non-invasive, non-thermal treatment utilizing small, cost-effective diode lasers [5, 6]. They are characterized by using low energy density parameters that have a photochemical effect on cells, imparting several biostimulatory impacts on target tissues [7]. These low-level parameters render PBM a safe intervention, largely free of potential side effects [8]. The underlying principle is that exposure to specific wavelengths induces a change in cellular phenotype, resulting in an increase in both the rate of cell proliferation and metabolic activity essential for wound healing [9].

Post-extraction healing begins with blood clot formation, progressing to granulation tissue and subsequently immature woven bone. Photobiomodulation therapy applied during this period actively enhances the necessary inflammatory cascade to promote tissue regeneration and osseous repair [10]. Studies have established that PBM induces structural alterations in neurons, inhibits fast axonal flow, and reduces the mitochondrial membrane potential. Collectively, this results in a blockade of the nerve signals, which consequently inhibits pain perception [11, 12].

Despite the increased use of photobiomodulation in all dental fields, its clinical efficacy in pain management remains controversial due to significant inconsistencies in the literature. Although some trials report reduced post-operative pain in children [13, 14]. A critical consideration in oral photobiomodulation is tissue optics and wavelength selection. While near-infrared wavelengths target deeper bone structures, the 660nm visible red wavelength matches the primary absorption peak of superficial mitochondrial cytochrome c oxidase and hemoglobin, making it highly effective for accelerating pediatric soft-tissue healing, stabilizing the fresh post-extraction coagulum, and targeting superficial nociceptors for rapid analgesia without risking excessive deeper thermal accumulation in young developing alveolar structures. [15] Recent systematic reviews suggest that these analgesic effects often fail to show a superior advantage over standard care [16, 17]. This lack of consensus necessitates further investigation into standardized therapeutic protocols. Therefore, the purpose of this study was to clinically evaluate the effect of photobiomodulation therapy at a wavelength of 660nm on enhancing wound healing and managing post-extraction pain in pediatric patients. The Null hypothesis is that there is no significant difference between using photobiomodulation therapy compared to post-extraction instructions in terms of wound healing and post-extraction pain.

### Study design and setting

This double-blind, split-mouth, randomized controlled clinical trial was conducted at the Department of Pediatric Dentistry and Dental Public Health and the Laser technology clinic, Faculty of Dentistry, Alexandria University, between June and October 2025.

### Ethical consideration

The trial received ethical approval from the Research Ethics Committee of Alexandria University, Faculty of Dentistry, on December 12, 2024 (IRB No. 001056—IORG 0008839) and was registered on ClinicalTrials.gov (NCT07033403) (https://clinicaltrials.gov/study/NCT07033403).

The research followed the Declaration of Helsinki [18], and was reported adhering to CONSORT 2025 and CONSORT PRO guidelines [19, 20]. Before enrollment, the parents or legal guardians of participating children were provided with information regarding the study’s objectives and protocols. The explanation detailed the nature of photobiomodulation therapy, the required use of protective eyewear, and the potential benefits regarding post-extraction pain management. Parents were assured that participation was entirely voluntary, that confidentiality would be strictly maintained through anonymized data coding, and that they retained the right to withdraw their child from the trial at any stage without any compromise to their ongoing dental care. Verbal assent and written informed consent from parents or guardians of eligible children were obtained at the start of the clinical trial.

### Sample size calculation

Sample size was estimated based on assuming 5% alpha error, 95% confidence level and 80% study power. The mean difference in wound size after 7 days was 4.42 mm^2^ for the laser group and 3.14 mm^2^ for the control group according to Özer & İnci (2024) [14]. Based on comparison of paired means and the highest SD = 1.77 to ensure enough study sample, a minimum sample size of 18 teeth was required per group, yielding an effect size of 0.723. The total required sample size = Number per group × Number of groups = 18 × 2 = 36 teeth in 18 patients. The sample size was based on Rosner's method, calculated by G*Power 3.1.9.7 [21, 22].

### Inclusion and exclusion criteria

This study included children aged 5—8 years, with Frankl Behavior Rating Scale (FBS) scores 3 and 4 assessed by a single, trained pediatric dentist during the screening process [23], free of any systemic disease or special health care needs (ASA I) [24]. Dentally, each child had at least one bilateral mandibular primary molar indicated for extraction with clinical signs and symptoms of pulp degeneration, such as sinus tracts, radiographic evidence of periapical or interradicular radiolucency, and/or non-restorable crowns [25, 26] Children were excluded from the study if they presented with: a history of allergy to local anesthesia; acute pain; systemic conditions such as prolonged bleeding, platelet disorders, hypersensitivity or known allergies to pain killers; or contradictions to laser therapy. Additionally, teeth exhibiting resorption exceeding one-third of the root length and primary molars exhibiting Miller’s Grade II or III tooth mobility were also excluded [27, 28].

### Blinding

Participant and parental blinding were ensured by providing a sham (placebo) laser application to the control side, during which only the aiming beam was activated. For consistency, the characteristic sound of the active laser was prerecorded and played during the sham procedure. The test side received the actual photobiomodulation intervention. In addition, both the outcome assessor and the statistician were blinded to the allocation of each side throughout the study.

### Randomization and allocation concealment

Eligible sides were randomly assigned to either the low-level laser group or the sham group using a computer-generated random sequence with a 1:1 allocation ratio (https://www.sealedenvelope.com/randomisation/) [29]. Randomization was performed by an independent researcher not involved in treatment or outcome assessment. Each child was assigned a unique serial number corresponding to the allocation list. The allocations were written on identical slips of paper and sealed in opaque, sequentially numbered envelopes [30]. The envelopes were stored and managed by a trial-independent member who was responsible for opening the envelopes only at the time of the local anaesthesia appointment.

### Clinical procedure

#### Preliminary screening visit

Full medical and dental history was taken from the selected patients whose parents had given their consent to participate [31]. Proper diagnosis by a thorough clinical and radiographic examination, intraoral periapical x-rays of the teeth to be extracted were taken to ensure that the patient matched the inclusion criteria. No treatment was done to the child during the first visit in order to build a strong patient-dentist relationship [32, 33]. Parents and children were given age-appropriate oral hygiene instructions, including proper tooth brushing twice a day, especially before bedtime, as well as flossing if indicated [34].

#### Extraction procedure

To ensure consistency, all clinical procedures were performed by the same pediatric dentist. For both groups, a 20% Benzocaine topical anaesthetic gel was used to obtund the discomfort associated with needle insertion. Subjects were told that their cheek will feel big and funny for some time. A standard IANB anesthesia (ARTINIBSA, Inibsa Dental S.L.U) was administered using the 27-gauge long anesthetic standard blade bevel needles at a rate of deposition of 1 ml/60 s. The disposable needle was directed at the level of the occlusal plane from the opposite side until bony resistance was reached. The needle was then withdrawn 2 mm to aspirate, this was supplemented by long buccal infiltration for the buccal gingiva. The amount of anesthetic solution administered was calculated according to the patient body weight [35]. One cartridge containing Articaine hydrochloride 4% with 1:100,000 epinephrine was used [36]. After achieving adequate anesthesia, extraction was carried out according to AAPD guidelines [37]. Lower full crown forceps or elevators or both were used, and care was taken to support the mandible with the non-extraction hand. A one-week interval was implemented between the extraction of the first and contralateral side as a washout period to optimize post-operative comfort and function.

#### Post-extraction intervention

Group I (test side) was treated, after extraction, with photobiomodulation therapy using a diode laser (SiroLaser Blue, Dentsply Sirona, USA) at a center wavelength of 660 nm operating in a Continuous Wave (CW) emission mode. The laser energy was delivered to the tissues via an 8 mm rigid MultiTip therapy light guide. The nominal beam spot size at the target was defined as 0.503 cm^2^, derived directly from the diameter of the tip. At a constant output power of 0.1 W (100 mW), this configuration yielded a precise power density (irradiance) of 0.20 W/cm^2^ at the tissue surface. The laser was applied for a total active irradiation time of 60 s per socket, divided equally into 20-s intervals along three distinct aspects: the facial, lingual, and occlusal surfaces. This application protocol resulted in an energy density (fluence) of 3.98 J/cm^2^ and a total energy delivery of 2 J/second treated surface, culminating in an absolute cumulative energy dose of 6 J per extraction socket. The laser was held in a stationary, non-contact position 3 mm away from the alveolar mucosa, tightly standardized using a calibrated periodontal probe guide. Due to this non-contact distance, minor geometric beam divergence naturally occurs, resulting in a slightly expanded true spot area at the tissue interface [14, 17].

For Group II (placebo side), no actual laser application was performed after the extraction of the contralateral primary molar; only the aiming beam was turned on (sham laser treatment), with the sound of the active laser played in the area using a mobile phone. The clinician, patient and guardian wore wavelength-specific protective glasses in accordance with standard laser safety guidelines [38]. Both groups were given post-extraction instructions. Children were told to bite on gauze with firm pressure against the surgical site for 30 min. The surgical site should not be disturbed. No vigorous rinsing should be done on this day. Post-extraction instructions also included avoiding scratching or injuring the cheek, lips, or tongue if numbness is felt. Cold soft food was recommended, as well as drinks to keep the child hydrated, but without using a straw [37]. A standardized rescue analgesia protocol was established; consisting of paracetamol suspension (15mg/kg per dose; maximum 4 times daily) or ibuprofen suspension (10mg/kg per dose; maximum 3 times daily) administered strictly on as-required basis for post-operative pain management [39]. Planning for space maintenance was considered.

### Outcome assessment

#### Wound healing assessment

Landry, Turnbull, and Howley (LTH) wound healing index was employed on days 3 and 7 post-extraction. Wounds were graded as follows: Grade 1 (Very poor), characterized by severe redness, bleeding, and no epithelialization; Grade 2 (Poor), exhibiting moderate redness and less than 50% margin epithelialization; Grade 3 (Good), showing mild redness and 50% or more epithelialization; Grade 4 (Very good), featuring healthy pink tissue with near-complete epithelialization and no bleeding; and Grade 5 (Excellent), representing complete epithelialization with healthy pink tissue and total wound closure. [40]

#### Post-extraction pain assessment

Post-operative pain was assessed using parts II, III, and IV of the Arabic Version of a self-reported questionnaire assessing pain, discomfort, analgesic consumption, daily activities, and related jaw-function impairment in children [41]. Part II, "4 items," was completed via a phone interview with the child on the first evening after the extraction procedure. Questions 1 and 2 in this part were about pain and discomfort from the extraction site, rated on a modified Wong-Baker FACES pain rating scale. Questions 3 and 4 concerned analgesic consumption. Part III, "13 items," was completed one week after the extraction and divided into three sections. The first, "2 items," were concerned with pain and discomfort from the extraction site after one week. The second section, "6 items," targeted analgesic consumption during the week after the extraction procedure, while the third section, "5 items," tackled changes in daily activities during the week after the dental extraction. Three questions required binary responses (yes/no), and two questions were open-ended. Part IV was also completed after one week and included a total of 10 items related to jaw function, with four items related to eating specific food types, two items related to psychosocial activities, and four items related to mandibular function. Both parts III and IV were completed at the scheduled one-week follow-up visits via printed paper.

#### Inter and intra-examiner reliability

Intra-examiner reliability was assessed by having the primary operator re-evaluate all scores and measurements after a two-week interval. The repeated assessments demonstrated excellent agreement for both the LTHI scores (κ ≥ 0.90). Inter-examiner reliability between the primary operator and the independent blinded outcome assessor was also excellent (κ = 0.86) [42].

#### Statistical analysis

Descriptive statistics were calculated as means, standard deviation (SD), median, interquartile range (IQR), frequencies, and percentages. Normality was assessed using descriptive statistics, histograms, Q–Q plots, and Shapiro Wilk normality test. As the data were not normally distributed, non-parametric tests were applied. The Wilcoxon signed-ranks test was used to compare measurements between the test and control sides. Changes over time within each group were assessed using Wilcoxon signed ranks test (Landry, Turnbull, and Howley wound healing index). Categorical paired data were analyzed using the McNemar test. Effect size (r) was calculated for Wilcoxon signed-rank tests using the formula r = Z/√N, where Z represents the standardized test statistic and N represents the number of paired observations. Effect sizes were interpreted as small (0.10), medium (0.30), and large (0.50). For categorical outcomes analyzed using the McNemar test, absolute risk differences (RD) were calculated to quantify the magnitude of treatment effects. Significance was set at *p*-value < 0.05. Data were analyzed using IBM SPSS Statistics for Windows, version 26.0 (IBM Corp., Armonk, NY, USA) [43].

## Results

**Fig. 1:**
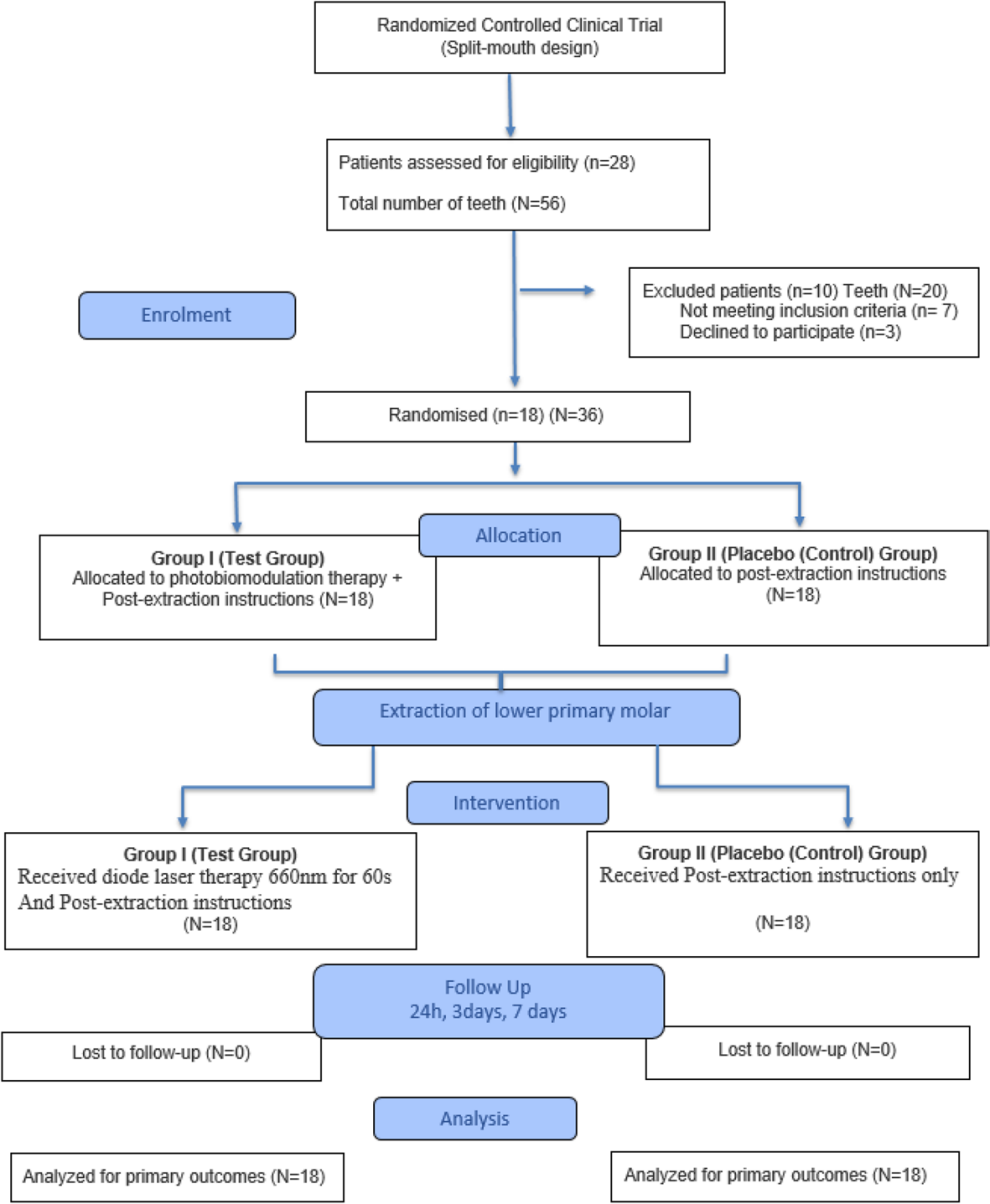
COSNORT flow diagram of participants throughout the study

A total of 36 teeth from 18 children were included in the study, with no loss to follow-up (Fig. 1). The children were 11 boys (61.1%), 7 girls (38.9%), with mean age 6.54 (± 1.04). Mandibular first primary molar was the predominant tooth extracted, accounting for 30 out of 36 teeth (83.3%); the remaining 6 teeth (16.7%) were mandibular second primary molars. (Table 1).

**Table 1:** Sample characteristics (*n* = 18)

**Age**	**Mean ± SD**	6.54 ± 1.04
	**Median (IQR)**	7.00 (5.65, 7.13)
Gender: n (%)	Male	11 (61.1%)
	Female	7 (38.9%)
Tooth: n (%)	D	15 (83.3%)
	E	3 (16.7%)
Frankl scale: n (%)	Definitely negative (1)	0 (0%)
	Negative (2)	0 (0%)
	Positive (3)	11 (61.1%)
	Definitely positive (4)	7 (38.9%)

*SD* Standard Deviation, *IQR* Interquartile range

**Fig. 2:**
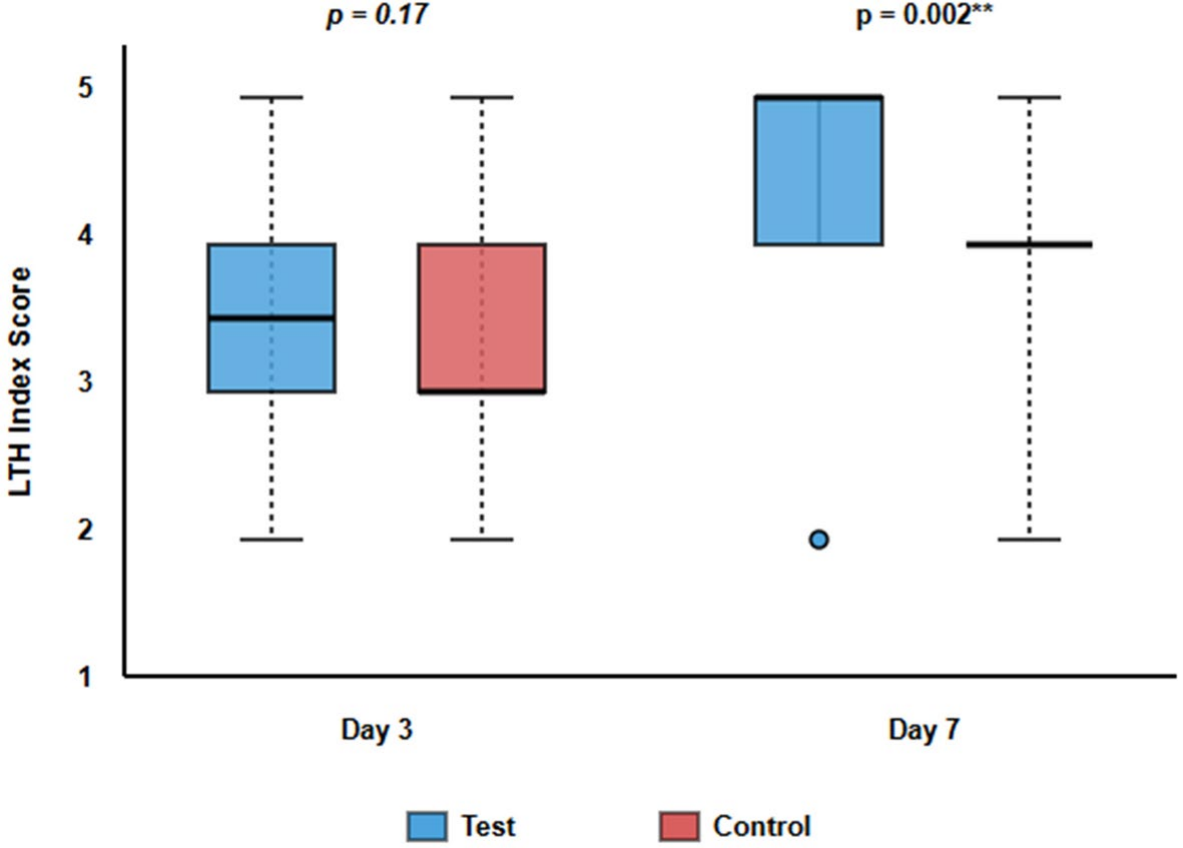
Landry, Turnbull, and Howley (LTH) in the two study groups across time

Table 2 presents Comparison of Landry, Turnbull and Howley (LTH) wound healing index between groups and across time showing no statistically significant difference between the groups at day 3 (Median: 3.00, *P* = 0.17), However, the test side achieved significantly superior wound healing scores by day 7 (*P* = 0.002*) (Fig. 2), reaching a median score of 5.00 compared to 4.00 on the placebo side

**Table 2:** Comparison of Landry, Turnbull and Howley (LTH) wound healing index between groups and across time

		**Test side**	**Control side**	**Between-group comparison**
Day 3	1	0 (0%)	0 (0%)	Z = 1.39
	2	4 (22.2%)	4 (22.2%)	P = 0.17
	3	6 (33.3%)	9 (50%)	r = 0.33
	4	5 (27.8%)	4 (22.2%)	
	5	3 (16.7%)	1 (5.6%)	
	Median (IQR)	3.00 (2.75, 4.00)	3.00 (2.75, 4.00)	
Day 7	1	0 (0%)	0 (0%)	*Z* = *3.13*
	2	1 (5.6%)	2 (11.1%)	*P* = 0.002*
	2	1 (5.6%)	2 (11.1%)	r = 0.74
	3	0 (0%)	2 (11.1%)	
	4	5 (27.8%)	11 (61.1%)	
	5	12 (66.7%)	3 (16.7%)	
	Median (IQR)	5.00 (4.00, 5.00)	4.00 (3.75, 4.00)	
Within-group comparison		*Z* = *3.14*	Z = 3.36	
		*P* = 0.002*	P = 0.001*	
		*r* = *0.74*	r = 0.79	

*IQR* Interquartile range

*Z* Wilcoxon signed-rank test statistic

*r* Effect size calculated as *r* = *Z/√N* and interpreted as small (0.10), medium (0.30), and large (0.50)

Between-group comparison: Wilcoxon signed-rank test comparing the test and control sides

Within-group comparison: Wilcoxon signed-rank test comparing Day 3 and Day 7 within each group

^*^Statistically significant at *P* < 0.05

**Fig. 3:**
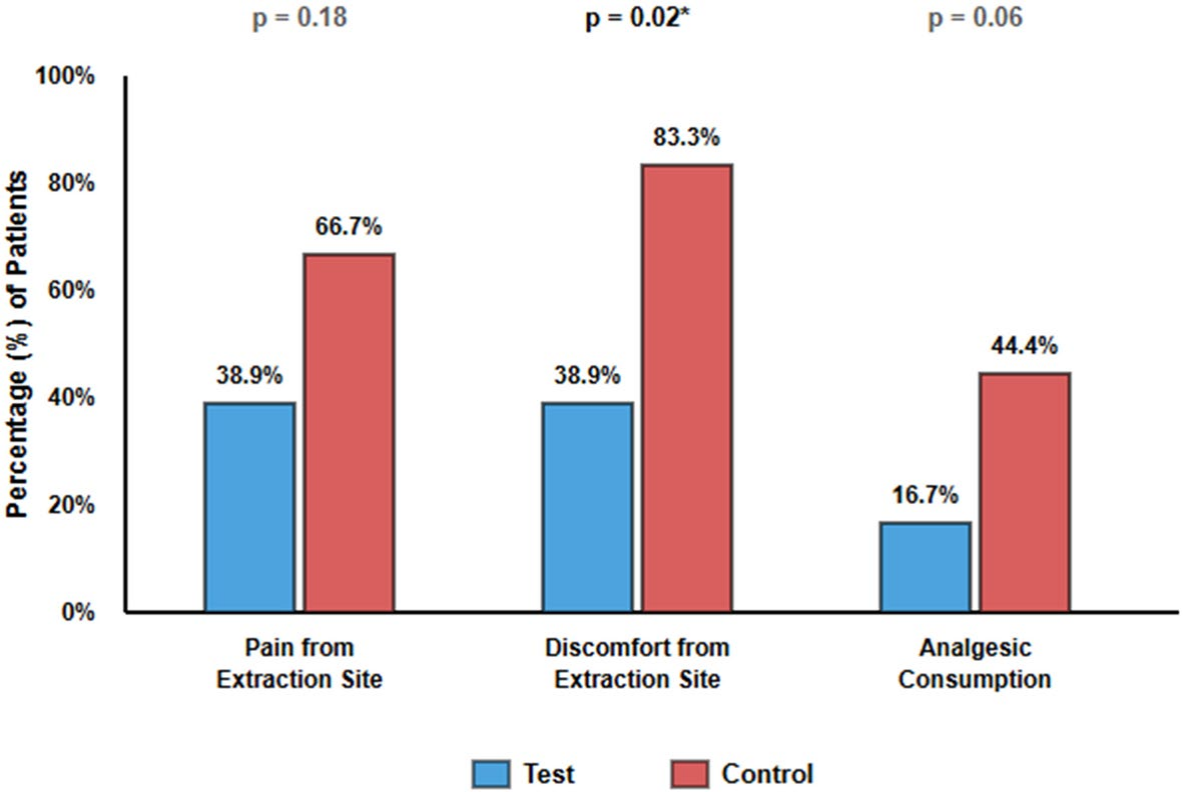
Pain and discomfort experience during the first evening after extraction in the two study groups (section II)

Figure 3 demonstrates pain, discomfort, and analgesic consumption during the first evening after extraction in the two study groups. PBMT group showed effective reduction in the subjective feeling of discomfort from the extraction site compared to the placebo side, which was statistically significant (*P* = 0.02*). It did not, however, differ significantly in the pain and analgesic consumption between the two groups in the first 24 h post-extraction (*P* = 0.18, 0.06).

Table 3 shows a comparison of post-extraction pain experience questionnaire one week after extraction (sections III and IV), showing significantly higher analgesic consumption over the week (33.3%) in the placebo group compared to none in the test group (*P* = 0.03*), all of which are non-prescription drugs, most commonly: Paracetamol and Ibuprofen. In comparison with the placebo group, the test group showed significantly less difficulty with chewing on the extraction site, chewing soft food and difficulty with taking a big bite compared to the placebo group; with percentages (38.9% vs.88.9%, *P* = 0.004*), (0% vs.33.3%, *P* = 0.03*), and (33.3% vs.72.2%, *P* = 0.04*) respectively.

**Table 3:** Comparison of self-reported questionnaire assessing pain, discomfort, analgesic consumption, daily activities, and related jaw-function impairment in children

			Test	Control	RD	*P* value
			N (%) yes			
1. Pain from extraction site			1 (5.6%)	6 (33.3%)	−27.7%	0.06
2.Discomfort from extraction site			6 (33.3%)	12 (66.7%)	−33.4%	0.07
3. Analgesic consumption	3.1 Analgesic consumption last week		0 (0%)	6 (33.3%)	−33.3%	0.03*
	3.2 Number of days using analgesics	1	-	3 (16.7%)	-	-
		2	-	2 (11.1%)		
		3	-	1 (5.6%)		
	3.3 Use of non-prescription drugs		0 (0%)	6 (33.3%)	−33.3%	0.03*
	3.4 Kind of analgesic	Cetal	-	3 (16.7%)	-	-
		Brufen	-	2 (11.1%)		
		Paramol	-	1 (5.6%)		
	3.5 Use of prescription drugs		0 (0%)	0 (0%)	0%	-
	3.6 Kind of analgesic		-	-	-	-
4. Daily activity	4.1 School absence		4 (22.2%)	10 (55.6%)	−33.4%	0.07
	4.2 Number of days	1	3 (16.7%)	8 (44.4%)	−27.7%	0.07
		2	1 (5.6%)	2 (11.1%)	−5.5%	
	4.3 Leisure activities refrain		2 (11.1%)	5 (27.8%)	−16.7%	0.38
	4.4 Number of days	1	1 (5.6%)	3 (16.7%)	−11.1%	0.37
		2	2 (11.1%)	2 (11.1%)	0%	
	4.5 Sleep disturbance		2 (11.1%)	6 (33.3%)	−22.2%	0.13
5. Jaw-function impairment after one week	5.1 Leisure		0 (0%)	1 (5.6%)	−5.6%	1.00
	5.2 Speech		0 (0%)	2 (11.1%)	−11.1%	0.50
	5.3 To take a big bite		6 (33.3%)	13 (72.2%)	−38.9%	0.04*
	5.4 To chew hard food		8 (44.4%)	13 (72.2%)	−27.8%	0.13
	5.5 To chew soft food		0 (0%)	6 (33.3%)	−33.3%	0.03*
	5.6 School work		2 (11.1%)	4 (22.2%)	−11.1%	0.50
	5.7 To drink		0 (0%)	2 (11.1%)	−11.1%	0.50
	5.8 To laugh		0 (0%)	0 (0%)	0%	-
	5.9 To chew on extraction site		7 (38.9%)	16 (88.9%)	−50%	0.004*
	5.10 To yawn		0 (0%)	1 (5.6%)	−5.6%	1.00

McNemar test was used

*statistically significant at *p*-value <0.05

*RD* Risk difference = percentage in test group − percentage in control group. Negative values indicate lower frequency of the outcome in the PBMT/test side

## Discussion

Modern dentistry utilizes various laser systems, most notably Diode and Er:YAG lasers, which are defined by their unique absorption characteristics and clinical applications. While Er:YAG lasers are frequently preferred for hard tissue procedures due to their high affinity to water and hydroxyapatite [44]. Diode lasers are widely valued for soft tissue applications and their proven efficacy in photobiomodulation therapy. The integration of laser technology into dental practice has revolutionized how clinicians approach tissue healing and patient comfort, utilized not only for specific procedures but as a general method for managing the common complications and oral disorders that follow routine dental treatments [8].

PBM offers numerous clinical benefits, primarily through its potent anti-inflammatory and analgesic properties [8]. It accelerates the physiological stages of repair by stimulating collagen synthesis, minimizing inflammatory exudates, and promoting both revascularization and epithelialization [6]. In addition, PBM effectively modulates pain perception by elevating endorphin release while simultaneously suppressing bradykinin levels [11]. This study compared the effect of 660nm diode laser application with placebo on mandibular primary molar extraction healing and post-operative pain, utilizing a split-mouth design to account for the subjective nature of pain perception**.** Results demonstrated that the photobiomodulation therapy group showed enhanced quality of wound healing by the 7th day compared to the control group, the post-operative questionnaire showed reduction in discomfort from extraction site in the first evening, lower analgesic consumption over one week post-extraction, and overall improvement in Jaw functions. Thereby the null hypothesis is rejected.

Oral wound healing is a complex process divided into overlapping phases: inflammation, proliferation, and remodeling. As day 3 typically corresponds to the peak of the inflammatory phase and the very beginning of the proliferative phase, the signs of inflammation (redness and swelling) may still mask the subtle initial improvements in tissue repair, leading to similar LTHI scores between the two groups as it measures the visible physical progress of healing [45]. In addition, the key repair cells (fibroblasts and keratinocytes) need time to significantly increase their proliferation, migration, and matrix production in response to the PBMT applied. By day 7, however, the inflammatory phase is resolving, and the proliferative phase is well underway. This is where PBM’s biostimulatory effects on cellular activities such as increased ATP production, enhanced cell proliferation, and angiogenesis are most pronounced and thereby clinically evident [8].These results are consistent with Astuti et al. (2022) [46] who reported histopathologically that the red diode laser (695nm) led to a substantial rise in lymphocytes, fibroblasts, and improved vascularization; crucially, it achieved deeper tissue penetration than the blue laser (403nm).

Although near-infrared wavelengths (810/980 nm) offer deeper penetration, the clinical efficacy of the shallower 660nm visible red light in mitigating deep pediatric socket pain is primarily driven by localized neuro-immunomodulation rather than a direct deep neural blockade. Initially, the 660nm wavelength is highly absorbed by cytochrome c oxidase within the superficial lamina propria and early blood coagulum, downregulating pro-inflammatory mediators like prostaglandins and Interleukin1β [15], which indirectly dampens peripheral nociceptor stimulation. This mechanism is uniquely amplified in pediatric patients, whose thinner oral mucosa and more porous alveolar bone allow a higher proportion of 660nm photons to reach the alveolar crest. Ultimately this accelerates superficial mucosal proliferation [17], establishing a stable biological seal that protects the underlying bone from mechanical irritation and significantly reduces subjective discomfort [13].

Girgis et al. (2021) [47] reported that 980nm PBMT was effective in managing pain level, swelling, and trismus after surgical extractions, which was confirmed by Le et al.(2022) using 810nm diode laser [48]. Given that wavelength availability differs regionally, our study utilized a 660nm laser, thereby furnishing new data regarding the clinical effectiveness of this specific wavelength. To the best of our knowledge, this study is the first clinical trial to use a validated self-reported Arabic questionnaire to evaluate the subjective, individual effect of photobiomodulation on post-extraction pain over 1 week [41]. Utilizing Patient-Reported Outcome (PRO) questions offers distinct advantages; they provide a standardized, objective view of the patient’s subjective experience. They uniquely capture the individual’s voice regarding symptoms, quality of life, and function, offering a comprehensive perspective often missed by traditional metric lab results. Furthermore, using validated questionnaires reduces bias and enables direct, meaningful comparisons of results across different studies and populations [49].

In a previous systematic review by Kulkarni et al. (2019) [10], laser therapy exerts significant neuropharmacological effects, increasing central neurotransmitters like serotonin while regulating peripheral inflammatory mediators such as prostaglandins. This chemical modulation is complemented by physical changes in the neurons; Amaroli et al. (2021) [50] noted that laser therapy directly interferes with nerve function by causing morphological changes in neurons, reducing their mitochondrial energy potential, and blocking fast axonal flow, which collectively results in a neural conduction blockade that effectively reduces the overall perception of pain.

These biological actions directly align with our clinical findings of the phone interview on the night of extraction, which showed a reduction in reported discomfort in the test group. This supports the premise that photobiomodulation therapy (PBMT) provides an immediate analgesic effect through nerve modulation and reduced edema, whereas healing acceleration is a subsequent, time-dependent process [51]. Interestingly, while reports of “discomfort” differed significantly, specific “pain from extraction site” scores and analgesic consumption did not show the same gap. This discrepancy may be explained by the difference between pain and discomfort concepts. While pain (nociception) is a specific, sharp, or throbbing sensation originating from nerve injury or inflammation, discomfort is a broader, global feeling of distress or unease. PBMT’s direct analgesic effect could have been overwhelmed on the first evening by residual local anesthesia or immediate post-operative analgesic consumption [52]. However, PBM successfully tackled the non-pain contributors to overall distress, such as swelling, fullness, and stiffness, resulting in a lower reported discomfort score. In addition, the difference in the self-reported outcomes of the first evening could also be due to the child being more familiar with the term discomfort, which is used more often than pain in pediatric dentistry.

Part III of the questionnaire tackled the week after extraction, 6 patients from the placebo group used analgesics, mainly Paracetamol and Ibuprofen; three children used analgesics for 1 day, two for 2 days, and only one child used it for 3 days post-extraction for pain relief, whereas regarding the test group, no children used analgesics for pain relief at all. Paracetamol and Ibuprofen carry distinct side effects, particularly in pediatric populations or when used repeatedly, such as hepatotoxicity, severe cutaneous adverse reactions (SCARS), gastritis, peptic ulcers, acute kidney injury, risk of thrombotic events, and antiplatelet effects [53, 54]. The fact that the test group had zero analgesic consumption highly suggests that the intervention managed discomfort and sustained that relief, eliminating the need for pills, hence eradicating the pharmacological risks, which provides a significant advantage in terms of patient safety and compliance. Part IV of the questionnaire, regarding Jaw functions, demonstrated that the PBM intervention provided superior functional capacity at the most critical site of injury, resulting in statistically significantly fewer patients who reported difficulty in taking a big bite, chewing soft food, and chewing on the extraction site, as well as overall improvement in all jaw functions, although nonsignificant.

Earlier studies investigating various PBMT wavelengths reported negative outcomes for pain control after primary molar extraction. Elbay et al. (2016) [13] reported no statistically significant differences in pain perception between the photobiomodulation and control groups following primary molar tooth extraction using the Wong-Baker FACES Pain Rating Scale (PRS) and the Visual Analogue Scale (VAS). Moreover, Özer & İnci (2024) [14] also reported no differences in post-operative pain using the same (PRS) scale immediately after extraction. Both studies used different laser parameters than our study. In addition, by restricting our analysis solely to extractions in the mandibular jaw, we successfully minimized or eliminated potential confounding factors that might have obscured the true effect of the intervention. Thus, our positive results highlight the importance of careful protocol standardization. This demonstrates that a targeted PBMT approach offers a dependable, non-pharmacological analgesic solution during the post-operative period.

Limitations of this study are the potential for a contralateral crossover effect characteristic of the split-mouth design, where behavioral conditioning or anxiety from the first extraction may have biased pain reporting during the second visit. Additionally, extraction difficulty was not objectively quantified using variables such as extraction duration, root morphology, or degree of physiologic root resorption. Although all extractions were performed by the same experienced operator following a standardized protocol, these factors may have influenced postoperative pain perception and wound healing outcomes. Furthermore, the non-contact laser application introduced minor beam divergence over the 3 mm air gap, potentially decreasing the true tissue-level irradiance and penetration depth compared to direct contact. Another limitation is that the visible light parameters used exhibit shallower penetration compared to near-infrared lasers. However, our positive clinical findings of both socket healing quality and post-operative pain management strongly validate the efficacy of our parameters. The reliance on the modified Wong-Baker FACES Pain Rating Scale remains a limitation of this trial, as it is an inherently subjective tool. Pediatric pain perception and reporting are heavily influenced by individual variations- such as baseline dental anxiety, cognitive maturity, and prior dental experiences- which can introduce personal bias and affect data reliability. Future trials should consider combining subjective scales with objective physiological markers to minimize these confounding factors. Multiple secondary outcomes related to analgesic consumption, daily activities, and jaw function were evaluated. Although exact *P*-values and effect size measures were reported, the possibility of type I error inflation should be considered when interpreting individual statistically significant secondary findings. Despite these limitations, the study demonstrated favorable effects of photobiomodulation therapy on socket healing and postoperative recovery. Given the promising findings of this study, Additional clinical trials are needed to evaluate different forms and settings of photobiomodulation therapy, specifically in pediatric patients. Future research could focus on parallel-group designs to eliminate any crossover effect. Although the use of CONSORT-PRO ensures transparent reporting of patient outcomes, the generalizability of these results may be limited by the highly controlled nature of the trial environment.

## Conclusion

Photobiomodulation therapy (PBMT) significantly enhanced wound healing by the 7th day post-extraction and immediately reduced patient discomfort. Crucially, it substantially reduced the reliance on analgesic consumption throughout the recovery week while significantly improving functional abilities. Therefore, PBMT is an effective adjunct therapy that accelerates recovery and improves the overall quality of life of children following extractions.

## Supplementary Information

Supplementary Material 1.

## Data Availability

All data included in this study are available from the corresponding author upon request.

## Abbreviations

PBMT: Photobiomodulation Therapy
LTH: Landry, Turnbull, and Howley
PEP: Post-extraction pain
IANB: Inferior Alveolar Nerve Block
LLLT: Low Level Laser Therapy
SCARS: Severe cutaneous adverse reactions
PRS: Pain Rating Scale
VAS: Visual Analogue Scale
